# Design-driven regional industry transformation and upgrading under the perspective of sustainable development

**DOI:** 10.1038/s41598-023-44190-8

**Published:** 2023-10-10

**Authors:** Lisi You, Tie Ji, Binbin Shao, Xiaojing Wu, Lei Shi

**Affiliations:** 1 College of Design and Art, Hunan University, Changsha, 410083 Hunan China; 2 College of Architecture and Art Design, Inner Mongolia University of Science and Technology, Baotou, 014010 Inner Mongolia China; 3 Baotou Garden Science and Technology Research Institute, Baotou, 014010 Inner Mongolia China; 4 Geological Survey Institute of Inner Mongolia Autonomous Region, Hohhot, 01000 Inner Mongolia China

**Keywords:** Community ecology, Sustainability

## Abstract

This study combines relevant theories and methods from economics and ecology to investigate design-driven transformation and upgrade paths for the long-term success of regional industries in the context of sustainable transformation, drawing on the design research literature's emphasis on sustainability, synergy, and a systemic approach. This evaluation may be thought of as a precondition for transformation. The regional industrial base dictates the upgrading route for sustainable transformation. Huaihua, a prefecture-level city in Hunan Province, China, serves as a case study for this investigation. Huaihua City's ecological footprints, human development index, and ecological welfare performance are used to evaluate the region's industrial base. A system based on quantitative data criteria and hierarchical analysis was built to choose top regional industries. The design study has promising implications for the sustainable transformation of major regional industries since it is an interdisciplinary, collaborative, and methodical type of research. Huaihua City, as a representative region of the less developed southwest region in China, designs a sustainable industrial transformation and upgrading path by providing a theoretical basis and quantitative measurement criteria for the sustainable transformation of regional industries.

## Introduction

Humans have paid high environmental costs in exploiting and modifying nature due to the fast rise of the world population and the quick development of science, technology, and industry^1,2^. The development stage with the gross domestic product (GDP) as the primary assessment target has shown us that the unlimited claim on natural resources, the obsessive pursuit of GDP, over-exploitation, and a series of economic-only development approaches typical of the crude quantitative growth of economic development have caused a severe waste of ecological resources and can no longer adapt to the idea of sustainable regional economic development^3^. Regional eco-industrial clusters are limited by the established industrial base^4^. Ecological civilization creation requires finding a synergistic regional economic, ecological, and socially sustainable development model. At the 1972 United Nations Seminar on the Human Environment, sustainable development (SD) was explicitly debated to address resource restrictions, severe environmental pollution, and ecosystem destruction^5^. In such a scenario, this research thinks that directing the growth direction of leading industries in the area and encouraging regional industrial structure rationalization would directly affect the region's future development. Government decision-making and company transformation route selection will depend on sustainable industry transformation pathways.

The UN conferences, "America by Design: Science, Technology, and the Rise of Corporate Capitalism," and "Design for the Real World" may have preceded design studies' emphasis on sustainable development. Design studies approach limited earth resources from a design viewpoint^6^. Integration, synergy, and practice characterize the design research. In terms of full life cycle design (LCA), eco-design, and green design, it has now interfered with the whole sustainable development process (Table 1)^7^. The former International Council of Industrial Design (ICSID), now the World Design Organization (WDO), redefined design: "The goal of design research is to improve people's experience of products, systems, services, etc. on a sustainable basis and to link innovation, technology, culture, economy, value, industry, and users in a sustainable development framework for regional industrial, economic, social and environmental domains to provide new value^8^." Design research can identify sustainable innovation opportunities by assessing user needs, new technologies, and sustainability issues. Design research can help build sustainable products and services by studying their environmental and social impacts. It may also reduce waste, boost energy efficiency, and sustain resource use.

Sustainable development can be accomplished through the use of design in a wide variety of new contexts and applications. The designers are able to:

- Create products and services that have a smaller negative impact on the environment and a greater rate of efficiency.
- To better withstand the consequences of climate change and other environmental pressures, more robust systems need to be developed.
- Improve people's living conditions as well as the conditions of their communities as a whole.
- Foster social equity in addition to environmental fairness as a primary goal.

**Table 1:** Comparative analysis of ecologically relevant design concept.

Design research methods	Targeted value and method
Green design	The green design emphasizes using materials and energy with low environmental impact. It proposes the 3R concept (Reduce, Recycle, Reuse), which means that the consumption of materials and energy is reduced, products and parts can be quickly sorted and recycled, and they can be recycled. At this stage, environmental issues were incorporated into the essential elements of design thinking for the first time, and the social value of design was significantly enhanced^9^
Ecological Design	Eco-design is a design method that covers the whole product life cycle process. It is not only focused on the final result but also considers the environmental issues in all phases and stages of product design and is considered an "in-process intervention." This is used to promote eco-design, which uses a systematic approach and quantitative indicators to guide and regulate the design process^10^
Sustainable design	At present, sustainable development has become the mainstream thinking of the international community and has a substantial positive impact. Sustainable design is a systemic design thinking, which includes not only the design of things but also the systematic interventions and process interventions of products and services from the perspective of service design^11^

Sustainable design has progressed from designing "things" to designing systems using these interventions from product, service, and system design research^12^. John Heskett states that design as a systemic approach can seamlessly span multiple levels within a system from production, consumption, innovation, and education, integrating culture, technology, economics, and ecology to create a complex mega-system of social, cultural, and economic relevance that bridges and regulates the expectations of inter- and intra-system participants^13^.

**Figure 1:**
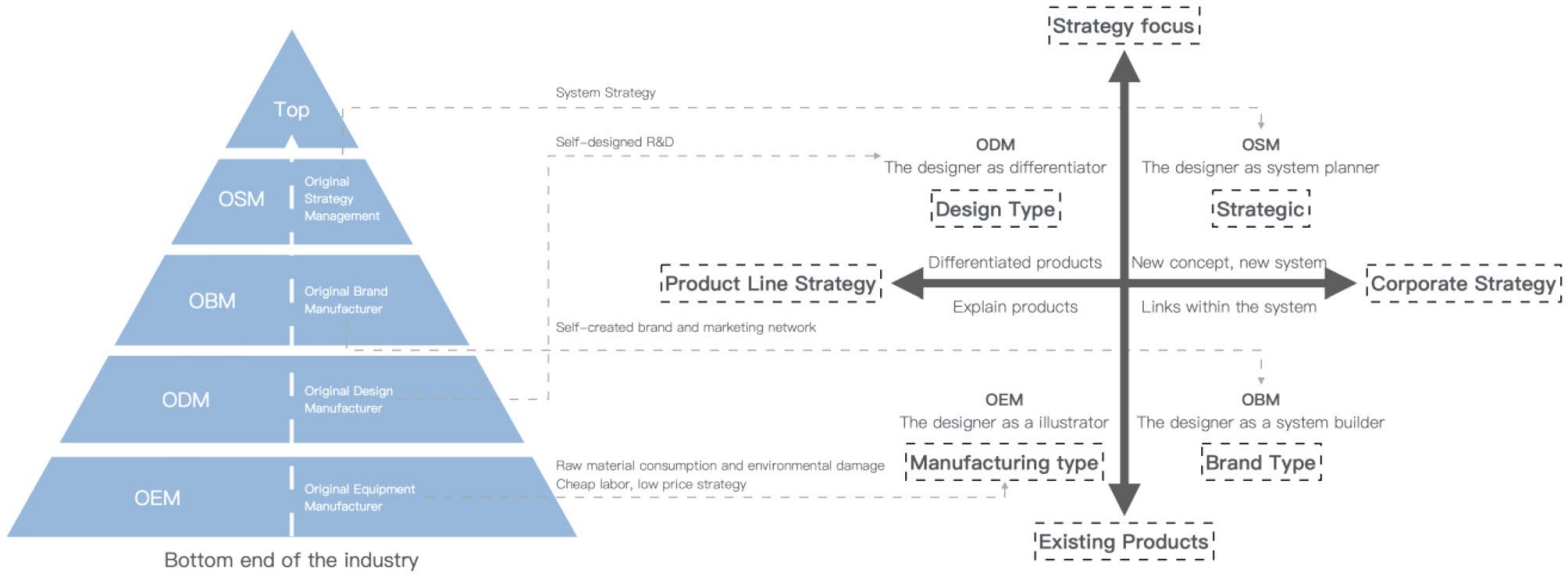
Different dimensions of design intervention industry.

By understanding the different latitudes of design intervention in industry and eco-industrial development, it can be found that there is a close correlation between the latitude of design intervention in industry sustainability and the specialized mode of production proposed by economist Adam Smith in his "Wealth of Nations" (as shown in Fig. 1), where: the OEM (Original Equipment Manufacture) model, in which the designer merely puts a firm or user's notion into practice and caters to the needs of the client; ODM (Original Design Manufacture) model, in which the designer identifies the market target by identifying the unique features of the product and offers the client complete services ranging from product creation, design, and manufacture to post-maintenance; OBM (Original Brand Manufacturing) model, in which the designer serves as the brand's central figure and all other areas of the industry supply chain, including R&D, production, and sales, are based on design; at this point, design starts to exhibit systemic features; OSM (Original Strategy Manufacture) model considers design research as a system-thinking research technique, where the designer may produce designs and integrate and build systems and management systems.

As shown in Table 2, various manufacturers have varied design intervention proportions. OEMs control design but make the least profit. ODMs earn more but have less design control. OBMs are the most customizable yet risky. OSMs have the lowest risk and control. The company needs to determine the appropriate producer for a product. An ODM may be suitable for a high-margin, low-risk product. OBMs may be suitable for high-risk, highly customized products. OSMs may be excellent for companies seeking low-cost, low-risk products.

**Table 2:** Different dimensions of design intervention in industry.

Dimension	OEM	ODM	OBM	OSM
Design responsibility	Original equipment manufacturer (OEM) designs the product and the original design manufacturer (ODM) manufactures it	The original design manufacturer (ODM) designs and manufactures the product, and the original brand manufacturer (OBM) brands it	The original brand manufacturer (OBM) designs the product and has it manufactured by a contract manufacturer (CM)	The open-source manufacturing (OSM) community designs and manufactures the product
Level of customization	Low	Low	High	High
Degree of control	High	Low	High	Low
Risk	Low	High	Low	High
Profit margin	Low	High	High	Low
Examples	Apple iPhone	Dell laptops	Nike shoes	MakerBot 3D printers

Huaihua, like other cities in China, has a poor industrial foundation and lagged growth^14–16^. The projected ecological civilization in China offers potential and problems for southwest cities. This study uses Huaihua as an example to combine ecological welfare performance, human development index, ecological footprint measurement, and other ecological and economic studies into sustainable design research^17–21^. The proposed mechanism of leading industry selection based on measurement results and hierarchical analysis and the construction of a regional industrial cycle system combining leading industries from the perspective of sustainable transformation will support the sustainable industrial transformation development of cities in southwest China.

Huaihua's case study could inspire industry reform by switching from agriculture and mining to renewable energy and IT. Huaihua may show regional industrial transformation successes. These include government funding, skilled labor, and business-friendly conditions. Huaihua's metamorphosis help regional industry upgrading in numerous ways. Huaihua case study also promotes investment, innovation, and market access. It shares knowledge and resources with other regions to boost competitiveness. Huaihua indicates regions may change their economies for sustainability. The Huaihua case study also encourages stakeholder involvement. In Huaihua; government, industry, and civil society have encouraged industrial change with the help of the success model, transformation variables, and transformation lessons that are offered.

Sustainable regional industry upgrading is hard which demands regional needs, resource availability, and political and economic conditions to be addressed. Regional industrial upgrading in Tech companies that are using sustainable resources consume less energy. Innovation and training can generate sustainable enterprises and increases employment gradually. Sustainability also requires environmental protection for industrial advances in regional areas. California heavily funds renewable energy and environmental preservation. California, Curitiba, and Brazil pioneered urban sustainability and renewable energy. Recycling, parks, and transit are Curitiba's top objectives are inspiring sustainable cities worldwide. China is spending heavily in renewable market. Singapore invested in green buildings with solar panels and rainwater collection. Europe's organic agriculture business, including food safety and sustainability, is also booming. Thus, upgraded regional industries supported sustainable growth to gain importance globally.

## Research region and methods

### Research region

#### Geographic location of Huaihua City

Huaihua City, the biggest prefecture-level city in Hunan Province, is situated southwest of the Wuling Mountains and Xuefeng Mountains. It's renowned as "the gateway to Qian and Yunnan" and "the throat of the Chu." The Xiangqian Railway, Zhiliu Railway, and Shanghai-Kunming Passenger Train converge near Huaihua, boosting its economy. The Shanghai-Kunshan Motorway, Baumao Motorway, and the newly reopened Zhijiang Airport have made Huaihua a major east–west Chinese transportation and logistics hub. Huaihua City's position boosts growth and radiation.

#### Ecological resource of Huaihua City

Huaihua is the "hometown of broad wood," "hometown of melons and fruits," and "hometown of medicinal herbs." Huaihua contains 11 GI goods, including Qianyang Ice Sugar Orange, Yuanling Tea, and Xinhuang Yellow Beef. Hunan Province's biggest tree accumulation volume is 86.96 million cubic meters in the city. 173.33 square km are planted with 1,900 Chinese herbs, including 175 national key-protected ones. Poria and asparagus lead national production. Huaihua has a 4.99 million-kilowatt hydro energy reserve and substantial wind and water resources for sustainable renewable energy. Renewable solid energy supports large electrical and green water sectors. It sustains Huaihua's ecological industry with high-quality production resources. Huaihua has 42.6% of Hunan Province's minerals, 48 types in 11 categories. Hunan Province has first, third, and fourth gold, copper, and phosphorous deposits. These high-quality mineral resources offer an industrial backbone for Huaihua but also pose significant ecological management, waste treatment, and post-mining ecological restoration issues^22^. How to break through the development bottleneck of resources and environment and realize the growth of ecological industries and mineral resources is the main dilemma for Huaihua to construct ecological industrial cycle development and create beautiful Huaihua.

#### Cultural resource

Huaihua, the cradle of Hunan culture, has rich cultural resources with high study, conservation, and development values. The Third National Cultural Relics Census found 1732 immovable cultural heritages, and 14 national and 33 provincial intangible cultural heritage conservation initiatives in Huaihua (Table 3). Huaihua and its surrounding areas have natural landscapes like Zhangjiajie, Used Mountain, Fanjing Mountain, and Xuefeng Mountain; human landscapes like Phoenix Ancient City, Hongjiang Ancient Shopping Centre, and Qiancheng City; red cultural tourism resources like Passage Conversion, Zhijiang War Surrendered City, and Xuefeng Mountain Anti-Japanese Battlefield; and the former residences of celebrities like Wei Yuan, Cai Yuan, and Cai Yu Cultural resources will become the embodiment of soft power in Huaihua's ecological civilization construction and help sustainably upgrade its industry in the construction of leading industries and other areas.

**Table 3:** Cultural resources in Huaihua City.

Name	Classification	Main tourism resource unit	Amounts
Folk Culture	National Intangible Cultural Heritage	Chashan Haozi, Chenhe Mulian Opera, Dong Nuo Opera, Chenhe High Tune, Dong Brocade Making Skills, Dong Lusheng, Yuanling Chenzhou Nuo Opera, Jingzhou Miao Song Song	14
	Provincial Intangible Cultural Heritage	Xupu Nuo Opera, Silkworm Lantern Dance, Dong Throat Song, Xuefeng Broken Neck Dragon Dance, Zhijiang Nielong Dance, Dong Style, Lianshan Bullfighting Dance, Chenxi Silk Strings, Zhijiang Mingshan Stone Carving, Chinese Bamboo Hats, Mayang Lantern Opera, Gan'ao, Fu's Woodcarving, Gongxi Yaojing Village Nuo Opera, Xinhuang Bullfighting, Thrushcross Fighting, Rolling Mud Fields, Changchang New Festival, Xupu Huayao Flower Picking, Lotus Lotus Field, Yuanling Folk Songs, Yuanling Competition Dragon boat, Xinhuang Eight Great Monsters, Nuo Opera "Gang Bodhisattva", Mayang Panhu Festival	33
Historical culture	Site type	Jingzhou Doupengpo Site, Hongjiang Gaomiao Site, Xinhuang Gaokanlong Site, Mayang Jiuqudujiang Bronze Mine Site, Yuanling Qianzhong County Site	5
	Attraction type	Longxing Temple, the first ancient temple in the south of the Yangtze River in the Tang Dynasty, Furong Tower, the first scenic spot in the upper reaches of southern Chu, the tomb of King Hou in the Yuan Dynasty, the tombs of the Western Han Dynasty in Huxi Mountain, the ancient Chinese houses in Jingping, the ancient village of Huitong Gaoyi, the ancient city of Hongjiang, the ancient city of Qiancheng , Ancient Dong Village	9
	Memorial type	Zhijiang Anti-Japanese Surrender Square, Tongdao Memorial Site, Xiangxi Bandit Suppression Monument, Xiang Jingyu's Former Residence, Su Yu's Former Residence, Teng Daiyuan's Former Residence	6
Humanities Culture	Allusions	"Scholarship Eryou", "Learn to be rich and five chariots", "Kuafu chasing the sun", "Yelang is arrogant", "Old and strong", "Horse leather shroud"	6
	Celebrity	Qu Yuan, Li Bai, Du Fu, Wang Changling, Wang Yangming, Lin Zexu, Shen Congwen, Qu Yuan, Xiang Jingyu, Mao Zedong, Teng Daiyuan, Yuan Longping, Su Yu	13
	Legend	There are legends about the Dong language in the Eryou Library Cave, Zhang Xueliang was imprisoned in Fenghuang Mountain, Ma Yuan was stationed in Pedou Mountain, Zhang Liang and Zhang Yu in Xinhuang, Panhu, the ancestor of the Miao nationality	5

Huaihua City has transformed and upgraded industries to support sustainable growth. Forests, rivers, and mountains surround Huaihua City and these resources developed forestry, agriculture, and tourism. Ceramics and silk manufacture showcase Huaihua City's rich cultural legacy. International visitors visit the city's historical landmarks. Huaihua City's educated workforce benefits the economy. Universities and research institutes in the city are developing new technologies and industries.

### Assessment method of regional industrial development level in the perspective of sustainable transformation

During ecological civilization building, regional industrial development levels determine regional eco-industrial cluster leaders. Comparing industrial architectures in east and west China, Gu suggested that eco-efficiency is linked to industrial restructuring^23^. Eco-efficiency and eco-welfare performance are frequently inseparable from industrial studies in economics^24^. Eco-efficiency is a GDP-based industrialization mentality aspect. However, eco-welfare performance incorporates the per capita ecological footprint and human development index (average life expectancy, education level, economic level), which indicates sustainable development throughout ecological civilization building.

Data were obtained without original data collecting. Secondary data, acquired by other organisations and made public, was all used. Officially, China's official statistics agency NBS^25^ provided Huaihua City's population data and land area. Regional GDP measures economic production. Understanding Huaihua City's ecological and industrial growth requires population, GDP, and land area statistics. Huaihua City's consumer market is based on population. These statistics help to understand Huaihua City's sustainability concerns and potential.

The National Bureau of Statistics of China (NBS), World Bank, and Global Footprint Network provided data for HDI and EF estimates. The HDI assesses human development in three dimensions:

- *Life expectancy*: The average number of healthy years a person is anticipated to live.
- *Education*: Average years of education.
- *Income*: Average income.

The EF is the quantity of biologically productive land and water needed to sustain a population or activity using current technology and resource management. It is commonly measured in global hectares (gha), which account for land productivity. In 2017, Huaihua City had a "medium" HDI of 0.767. Huaihua City has modest human development. Though the city's HDI matches Hunan and Jiangxi Provinces. Huaihua City's 2017 ecological footprint (EF) was 2.7 gha/person. Huaihua City now uses 2.7 times more land and water than it needs. This high EF shows the city's development practises are unsustainable. Huaihua City has a reasonable HDI and EF, however its development practises are unsustainable. Sustainable growth requires the city to lower its EF. The HDI and EF are imprecise measurements of human progress and sustainability. The HDI ignores inequality and environmental quality. The EF ignores land and water quality. Huaihua City has a "medium" HDI, which is decent for China. Renewable energy, energy efficiency, and trash reduction may help the city achieve this.

In this paper, population size, GDP, population education level (average years of education), population age structure (life expectancy), various types of land area, and data related to primary, secondary, and tertiary industries in Huaihua City are searched and analyzed using the human development index and ecological map footprint.

#### Human development index (HDI)

Human Development Index (HDI) measures population well-being. Life expectancy, education, and income determines it. The HDI measures Huaihua City's ecological and industrial sustainability by taking into consideration both social and economic factors. The HDI measures well-being comprehensively, life expectancy, education, and income are the HDI's well-being factors. This makes it a better well-being indicator than GDP per capita. Huaihua City's ecological and industrial growth may be compared to other cities using the HDI, a common statistic. It is helpful for assessing policies and programmes' well-being effects. Huaihua City can measure its sustainability progress and identify areas for improvement using the HDI. The HDI does not account for environmental quality or social fairness, making it a flawed measure of well-being. This might reveal areas for improvement. Huaihua City may utilise the HDI to track its sustainable development by considering these aspects.

The HDI consists of three dimensions:

- Health and health care (Longevity Index, measured by life expectancy at birth)
- Education (Education Index, measured by average years of schooling)
- The ability to live a decent life (Income Index, measured by GDP per capita)

The composition of the HDI shows that it focuses on improving people's quality of life and well-being, while the goal is to enable people to live long, healthy, and creative lives. The HDI has 0–0.549, 0.550–0.699, 0.700–0.799, and 0.800–1.000 as the low, medium, high, and very high-level classification intervals, respectively. The formula is:

$$HDI = \sqrt[3]{{Longevity\,{ }Index \times Education\,{ }Index \times Income\,{ }Index}}$$

$$Longevity\,{ }Index = \frac{{LE - LE_{min} }}{{LE_{max} - LE_{min} }}$$

$$Education\,{ }Index = \frac{{MYS - MYS_{min} }}{{MYS_{max} - MYS_{min} }}$$

 

$$Income\,{ }Index = \frac{{\ln \left( {GNI_{pc} } \right) - \ln \left( {GNI_{pcmin} } \right)}}{{\ln \left( {GNI_{pcmax} } \right) - \ln \left( {GNI_{pcmin} } \right)}}$$

The minimum value of life expectancy at birth ($${LE}_{min}$$) is 20 years, and the maximum value ($${LE}_{max}$$) is 85 years; the minimum value of expected age of education ($${MYS}_{min}$$) is 0 years, and the maximum value ($${MYS}_{max}$$) is 18 years; the minimum value of national income per capita ($${GNI}_{pcmin}$$) is US$100 and the maximum value ($${GNI}_{pcmax}$$) is $75,000.

**Figure 2:**
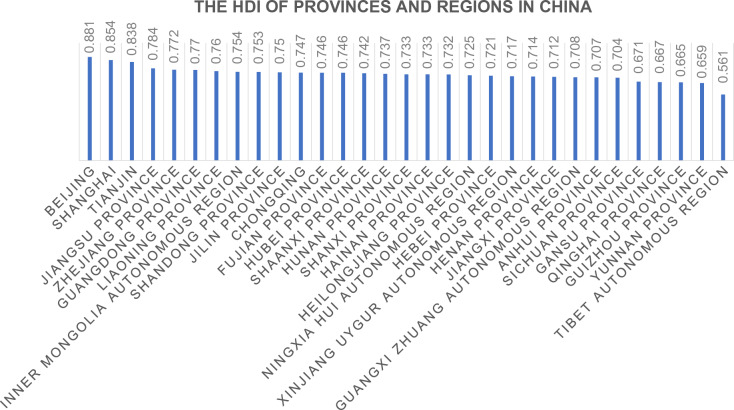
The HDI of provinces and regions in China, 2017.

China's HDI increased from 0.410 to 0.752 from 1978 to 2017, making it a high-human development country, according to the UNDP's Special Edition of the China Human Development Report 2020. Since the UNDP started measuring HDI internationally in 1990, China has been the only country to leapfrog human development^26^. Figure 2 shows 2017 Chinese provincial and autonomous region human development indexes.

Most of China, including Hunan Province, has a high HDI. However, subdivided provinces and regions have unequal regional human development indexes. In this paper, using 2017 data from Huaihua City, the HDI has steadily improved, with life expectancy per capita increasing from 71.5 years in 2000 to 75.5 years in 2017, average years of education increasing from 6.35 years to 10.5 years, and GDP per capita reaching $5050. Using the relevant HDI measurement formula, Huaihua City's HDI was 0.66 in 2017, still below the medium-level index of 0.699 and the only area in Hunan Province still in the medium index.

#### Ecological footprint (EF)

Ecological footprint (EF) is the quantity of biologically productive land and water needed to sustain a population or activity using current technology and resource management. It is commonly measured in global hectares (gha), which account for land productivity. EF needs data on several parameters, including resource use, waste output, and land productivity. Estimating EF for a city is challenging since local statistics are seldom accessible. A resource consumption and waste generation model may estimate EF for a city after calibration using data from other cities or areas. Estimating the productivity of various kinds of land in Huaihua City is challenging due to a paucity of data on resource use and trash output. Tracking EF helps the city detect resource and waste management issues. As data and methodologies improve, EF estimation continues. EF estimates may compare cities' resource utilization and waste management. This may assist the city discover sustainable development gaps.

Sustainability economists have seen the EF as the most complete metric of human natural consumption or ecological impact since Rees (2001) introduced it^27^. The EF shows the ecological threshold for human survival, and the higher the value, the more natural human consumption^28,29^. The WWF and World Bank calculate ecological footprint and carrying capacity as follows^30,31^:

$$EF = N \times ef = N\sum A_{i} \times EQF = N\sum \frac{{C_{i} }}{{P_{i} }} \times EQF$$

$$EC = N \times ec = N\sum A_{i} \times YF \times EQF$$

where “$N$” represents the total population, “$ef$” represents the ecological footprint per capita (hm^2^/person), “$i$” represents the type of consumption items, “${A}_{i}$” represents the actual ecological area occupied by “$i$” consumption items per capita, “${C}_{i}$” represents the per capita consumption of species consumption items, “${P}_{i}$” represents the average world production of “$i$” consumption items, “$EC$” represents the ecological carrying capacity per capita, “$YF$” represents the yield factor, and “$EQF$” represents the equilibrium factor, the equilibrium factor is due to the different productivity of land for different biological production types. And the yield factor varies by land use type and year, explaining the different productivity levels of specific land use types in different countries or regions, with national or regional characteristics.

A WWF-defined high-consumption region has an ecological footprint larger than 2^32^. The WWF, World Bank, and other agencies' ecological footprint assessment only measures to the country level, and the data is only updated to 2017. The National Footprint and Biological Carrying Capacity Account 2021's 2017 public data package elaborates on China's ecological footprint indicators: In 2017, China's per capita GDP was $1539, its biological carrying capacity was 0.9 gha, and its ecological footprint was 3.6 gha, making it a high-consumption ecological deficit nation. In 2017, Hunan Province's ecological footprint was 2.61 gha. After subtracting 0.899 gha of per capita ecological carrying capacity, the per capita ecological deficit is 1.718 gha, making it a significant ecological deficit region in China. However, the ecological footprint measurement result of Huaihua City appears to be exceptionally excellent, relying on the more abundant local water conservancy and wind power generation resources, the per capita ecological footprint is only 0.74 gha, while the per capita ecological carrying capacity is 1.79 gha, leaving a surplus of 1.05 gha, making it one of the rare areas with low ecological footprint consumption^33^.

#### Ecological welfare performance index (EP)

Herman et al.^34^, sustainable development economists, developed Ecological wellbeing Performance (EP) and Zhu et al. applied it to assess ecological wellbeing in China. Ecological welfare performance is the relationship between welfare and ecological resource consumption^35^, reflecting the level of well-being that natural materials can bring to humans and better reflecting the results of the ecological economy and ecological industry after optimization and upgrading^36^. Sustainable development requires maximizing human well-being with minimum ecological use. Quantifying eco-welfare performance is:

$$EP = \frac{HDI}{{EF}}$$

### Hierarchical analysis method—regional industry selection mechanism in the perspective of sustainable transformation

The Analytic Hierarchy Process (AHP) analysis uses pairwise comparisons of all indicators. Experts' subjective assessments determine the indicators' relative value^37^. AHP is a decision-making method that can be used to create a ranking assessment tool. AHP helps decision-makers by dividing tough decisions into criteria and sub-criteria. The decision-maker grades the two criteria from 1 to 9, with 1 equal relevance and 9 excessive significances. Pairwise comparisons create weight matrices where the weights show significance and rank options. In AHP criteria the meaningful and exclusive decision criteria are needed. AHP results may be affected by criteria weights.

AHP is a multi-criteria decision-making strategy for ranking choices. It helps choose regionally successful industries. It may rank industries by economic growth, environmental impact, or social advantages. The AHP may also identify and weight the most essential industry selection criteria. It is a methodical, transparent approach for objectively assessing possibilities. AHP can solve multi-criteria decision-making issues. Several studies have chosen regional development industries using the AHP. The World Bank selected industries for development in Vietnam's Mekong Delta using the AHP. The AHP helped the research identify regionally successful industries.

For transparency and adherence to standards experts may be selected by a panel. There should be enough specialists to represent different viewpoints. In this work we mentioned about 12 experts, but there might be more or fewer.

In this research, 12 experts from leading Chinese universities in ecology, economics, and agriculture rated the three criterion-level indicators using the Saaty 9-level scale^38^. The comparison judgment matrix $A$ is established based on the individual preferences of experts, the ${a}_{ij}$ represents the relative importance of ${a}_{i}$ to ${a}_{j}$, as determined by the experts. The dimensions of matrix $A$ are both $M$ and $N$, which represents the indexes in the criterion layer^39^.

$$A = \left( {\begin{array}{*{20}c} {a_{11} } & \cdots & {a_{1j} } \\ \vdots & \ddots & \vdots \\ {a_{i1} } & \cdots & {a_{ij} } \\ \end{array} } \right)_{M \times N}$$

The weight calculation is done under a single criterion.

$$\overline{{H_{i} }} = \frac{{\left( {\mathop \prod \nolimits_{j = 1}^{n} a_{ij} } \right)^{\frac{1}{n}} }}{{\mathop \sum \nolimits_{i = 1}^{n} \left( {\mathop \prod \nolimits_{j = 1}^{n} a_{ij} } \right)^{\frac{1}{n}} }}\left( {i = 1,2,...,n} \right)$$

The index weight vector obtained by the geometric average method is used to calculate the relative weight of index $i$ for each expert. After calculation, the normalized feature vector is obtained, and the maximum eigenvalue is calculated. Next, a new matrix is constructed using the scores of 12 experts.

$$\overline{C} = \left( {\begin{array}{*{20}c} {c_{11} } & \cdots & {c_{1j} } \\ \vdots & \ddots & \vdots \\ {c_{i1} } & \cdots & {c_{ij} } \\ \end{array} } \right)_{M \times N} \left( {i = 1,2,3;{ }j = 1,2,...,12} \right)$$

In matrix $\overline{C }$, the $i$ represents the weight of the primary indicators, while $j$ represents the experts. Finally, the scores of all 12 experts are integrated, and ${c}_{1}$, ${c}_{2}$, and ${c}_{3}$ represent the weight of the Human Development Index (HDI), the Regional Industrial Optimization and Upgrading Index, and the Ecological Industrial Index, respectively.

Consistency checks are then performed to ensure the accuracy of the judgment matrix. The value of the consistency index $CI$ is calculated using the formula:

$$CI = \frac{{\lambda max^{n} }}{n - 1}$$

With a smaller $CI$ means a higher consistency of the judgment matrix. The consistency index reflects the rigorous logic of subjective judgment. The random index $RI$ is introduced to eliminate the difference caused by the order of the matrix. Specific reference values of 0.00, 0.00, 0.58, 0.90, 1.12, and 1.24 are used for the order from 1 to 6. Finally, the final consistency ratio ($CR$) is calculated using the formula:

$$CR = \frac{CI}{{RI}}.$$

If the $CR$ is less than 0.1, the consistency test is passed. The $CR$ value of 0 indicates a perfect level of consistency in the pairwise comparison, while a $CR$ value greater than 0.1 indicates the need for revision in the judgment matrix.

## Results and discussion

The region-specific demands, resource availability, politics, and economy must be considered before applying them to any regional industrial reform. Huaihua's outcomes reflected local circumstances. Before applying the findings, examine whether they apply elsewhere. Huaihua's findings may need to be tailored to various places. Huaihua's findings may need to be adjusted for other areas' economies. The Huaihua case study may help southwest Chinese towns to grow sustainably. The government strongly supports education, training, and high-tech industry growth in Huaihua. Huaihua encourages innovation and investment in business. This attracts firms and creates employment. Huaihua's government, enterprises, and civil society collaborated to assist industrial change. Huaihua shows a region's economy that may be altered for sustainability. Huaihua prioritises environmental conservation as well. Environmental protection has increased residents' quality of life. Huaihua's example may help other towns build sustainable economies.

When implementing industrial transformation models to Huaihua City, socio-political environment is crucial. Huaihua's government has supported industrial change. It's crucial to understand government backing and ensure that suggested models match government aims. In terms of resource availability land, labour, and capital may impact model feasibility. Before choosing a model, it is crucial to understand the political and economic climate and ensure that suggested models can adjust. Huaihua City's socio-political situation changes keep up with Huaihua City's socio-political trends. To succeed, industrial transformation models must be adapted to Huaihua City's socio-political setting and should be carefully monitored and assessed.

The city has brought together government, business, university, and civil society leaders. Sustainable development stakeholders may cooperate on this platform. The city funds stakeholder-developed sustainable development initiatives. The city helps stakeholders build sustainable development initiatives. This support ensures successful and sustainable project implementation. Public education, media outreach, and stakeholder engagement raise awareness of sustainable development in the city. This helps stakeholders understand sustainable development and commit to working together to achieve it.

Design-driven techniques may encourage sustainable development via industry transformation and upgrading in other areas or nations. Industry, government, and other stakeholders may help design-driven initiatives succeed. This guarantees that the strategy meets regional or national requirements and has decision-maker support. Design-driven techniques should be executed systematically by beginning with identifying the issue. This requires changing manufacturing, generating new goods, or building new business models. Stakeholders should be informed about design-driven results. This will promote the technique and encourage replication.

### Assessment of the level of territorial development of Huaihua City in the context of ecological civilization

**Figure 3:**
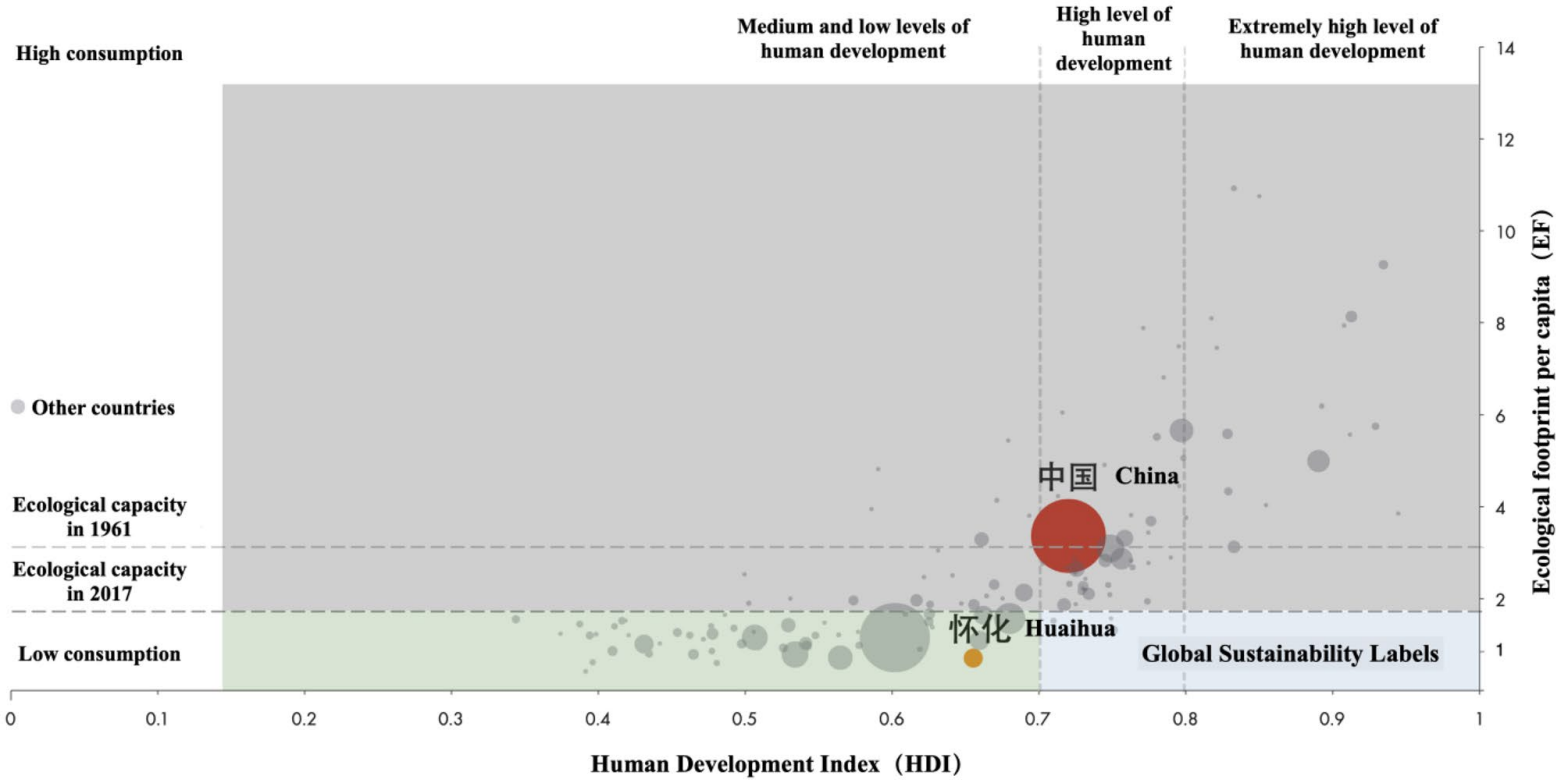
Ecological welfare performance index of Huaihua City and China.

According to the HDI, EF, and global ecological welfare performance distribution map (Fig. 3), Huaihua City is a low-income area with moderate welfare, good ecological resource preservation, and weak industry and human settlement development. Thus, when considering regional industrial upgrading, we should promote ecological and sustainable industrial development and consider whether it can boost human well-being indices like income, education, and life expectancy. These are important considerations for design-driven, sustainable regional industry transformation and upgrading.

Huaihua has high-quality manufacturing resources to maintain its ecological industry. Huaihua has a long history of farming with fertile agricultural region. Huaihua lies in a river basin with several rivers and streams. This guarantees irrigation and industrial water. Huaihua has extensive growing seasons which is perfect for growing several crops. Huaihua's workers have experience in agriculture, industry, and other fields. This makes it attractive to eco-industry investment. With good soil and a warm temperature, Huaihua is ideal for organic farming. With a trained workforce and a good climate, Huaihua may attract green industrial investment. Eco-friendly production utilises less energy, materials, and pollutants. Huaihua can improve its residents' future by growing these green enterprises. Sustainable management is needed to preserve Huaihua's high-quality production resources. Government policies must assist Huaihua's eco-industries. This includes financial incentives, infrastructural development, and favorable regulations for ecological industries in Huaihua.

### Design-driven upgrading path of rural eco-industry in Huaihua City

Three design-driven transformation approaches for regional industries are examined using economics and ecology theories and methods:

1. It examines economic growth and environmental sustainability using ecological economics. Ecological economics studies the economy-environment link. It claims that the economy is a subsystem of the environment and should be sustainable.
2. It analyses regional industry material and energy fluxes using industrial ecology. Industrial ecology studies industry-environment connections. It proposes greening industries.
3. It identifies and implements design-driven regional industry transformation routes using design thinking. Design thinking solves complicated challenges with creativity and empathy. It helps find creative, sustainable solutions for sustainable development.

These ideas and approaches are used to investigate regional industry design-driven transformation trajectories. It claims that design can generate sustainable goods and services and boost industry efficiency. The paper also lists some obstacles to design-driven regional industry development. Business, government, public cooperation, and R&D investment are among these problems. Businesses, government, and the public benefit from the research. Businesses and governments may utilise the data to discover sustainable innovation possibilities and build sustainable development rules and regulations. The results may help people comprehend sustainable development and demand sustainable goods and services. Design-driven regional industry change requires collaboration. Businesses, government, and the public must collaborate to find and execute these routes.

Designing sustainable products and services helps lessen the city's economic effect on the environment. Design may increase the efficiency of current industries, reducing the city's resource use. Design may foster cooperation between corporations, government, and the public to solve the city's socioeconomic issues. Huaihua City's case study shows regional industries' design-driven transformation potential. Design can solve many problems, and the case study gives a starting point for future investigation.

#### Basis for selecting the leading industry in the eco-industry

Selecting top industries supports regional economic growth and industry reorganization and upgrading^40^. According to American development economist Rostow, leading industries should be able to obtain new production functions through technological progress, have continuous high growth, and have a strong diffusion effect^41^.

Walt Whitman Rostow's^41^ five-stage economic growth theory covers the shift from traditional to contemporary economies. Stages are:

1. *Traditional society*: Subsistence agriculture, little division of labor.
2. *Pre-conditions for takeoff*: Market economy, stable government, and trained labor are developed in this stage.
3. *Takeoff*: Investment in capital goods and infrastructure drives fast economic expansion.
4. *The drive to maturity*: The economy grows as it diversifies and matures.
5. *The era of high mass consumption*: Affluence and consumption characterise this epoch.

Rostow's argument is oversimplified and ignores government policy's impact on economic development. It remains a popular foundation for economic growth.

Huaihua City illustrates Rostow's idea. Huaihua City remained traditional until the 1980s when its economy grew rapidly. Infrastructure investment, market economy development, and opening the Chinese economy to outside investment drove this expansion.

Due to its quick economic expansion, Huaihua City is currently called a "pre-mature" economy. Environmental deterioration and inequality plague the city. Huaihua City must solve these issues to grow sustainably.

Rostow's hypothesis has been implemented in many nations with varied degrees of success. The hypothesis works best in nations with robust markets and stable governments like the US. The idea has failed in nations with diverse traits, such as those with a history of political instability or those primarily reliant on natural resources. Rostow's theory ignores government policy's impact on economic development. Government policy may boost economic development, however. Government measures like infrastructure and education investment may set the stage for economic development.

The 1987 Brundtland Report defined sustainable development. This report defines sustainable development as the development that meets the needs of the present without compromising the ability of future generations to meet their own needs. The report lists sustainable development principles:

- Sustainable development must benefit everyone, regardless of socioeconomic level.
- Sustainable development must preserve future generations' natural resources.
- Sustainable development must be resource-efficient and waste-free.

Sustainable design requires long-term thinking. Monitoring and evaluating sustainable development programmes helps ensure that they are successful and addressing the needs of the people they are meant to aid. The three concepts of leading industries—obtaining new production functions, continuing high growth, and great diffusion effect—are explained below:

- Innovative companies may add production functions. Innovation might include new goods, processes, or business models. A top industry may produce more effectively and cheaply with innovative production functions. This may boost industrial profitability and cut consumer costs.
- Market share allows leading industries to develop continuously. They may give customers more appealing goods and services than other sectors. Reinvesting earnings in R&D helps leading industries to expand quickly and innovate.
- Leading industries' technology and breakthroughs are typically adopted by others, creating a powerful diffusion effect. As other sectors become more productive and efficient, economic development may result. Diffusion may also create new employment and markets.

Considering the assessment results of ecological welfare performance, the study area's leading ecological industries should focus on industries that can improve the human living environment, local residents' happiness and well-being, and sustainable synergistic development among regional ecology, economy, and society, while maintaining the current ecologies. This research analyses and aggregates regional economic development statistics based on the study area's industrial and urban growth's ecological welfare performance. This work analyses and researches industrial restructuring in Huaihua City from a sustainable development viewpoint using data from 2015–2019 owing to episodic occurrences affecting relevant data after 2020.

In Table 4, Huaihua City's GDP grew about 8% annually from 2015 to 2019. By 2019, the city's GDP was 58% tertiary. Special agriculture, forestry, animal husbandry, and fishery breeding still rely on agricultural grain crops, but herbal medicine, fruit, and vegetable cultivation, as well as cattle and pig breeding, are growing (Table 5).

**Table 4:** The main industry output value of Huaihua city in 2015–2019 (unit: RMB billion).

Year	GDP	Featured agriculture, forestry, animal husbandry and fishery	Industry and construction	Tertiary industry
2019	1616.64	399.1	448.26	943.86
2018	1484.54	330.73	425.64	869.9
2017	1377.31	350.31	400.73	790.78
2016	1347.54	336.47	394.63	772.29
2015	1256.28	309.43	383.76	703.81

**Table 5:** Output value of agriculture, forestry, animal husbandry and fish industries of Huaihua city in 2019 (unit: RMB billion).

Industries	Output value
Total Output Value of agriculture, forestry, animal husbandry and fishery	39,910.16
Agricultural	
Total	20,012.69
Paddy	5142.90
Vegetables (including vegetable melon)	6115.19
Fruit	4166.27
Chinese herbal medicine	1179.40
Forestry	
Total	3294.46
Bamboo harvesting	1513.76
Forest products	1581.19
Animal husbandry	
Total	14,366.75
Cattle feeding	1194.18
Pig breeding	10,195.85

The secondary economy, encompassing manufacturing and construction, accounts for 27.7% of the city's GDP, but 47% of its energy use (Table 6). Mineral resource development, the paper industry, and other secondary sectors use the most energy. New industries like pharmaceutical, agricultural, and by-product, and computer and electronic equipment manufacturing increase GDP while reducing local energy consumption, pollution, and consumption (Table 7).

**Table 6:** Energy consumption status of Huaihua City in 2019.

Index	Energy consumption (2019)	Percentage of energy consumption
Total energy consumption	714.15	100.00
Agriculture, forestry, animal husbandry and fishery	36.20	5.07
Industry and construction		
Total	334.97	46.90
Industry Total	319.77	44.78
Designated industries	278.53	39.00
Industries under regulation	41.24	5.77
Construction industry	15.21	2.13
Service industry		
Total	205.22	28.74
Transportation industry	129.93	18.19
Residential life		
Total	137.76	19.29
Cities and towns	87.16	12.20
Rural	50.61	7.09

**Table 7:** Energy consumption status of major industries in Huaihua City.

Industry	Comprehensive energy consumption (tons)	Total water intake (1 million cubic meters)	Gross industrial output value (RMB million)
Total	1,697,473	208.7038	99,108.82
Production and supply of electricity, heat, gas and water	256,813	179.1185	24,406.76
Non-ferrous metal mining and dressing industry, ore dressing, smelting and rolling processing industry	67,843	4.2545	15,588.24
Agricultural and sideline food processing industry (food manufacturing, wine, refined tea)	99,182	1.4136	15,240.68
Mining and dressing of non-metallic minerals, product industry	503,551	2.2821	14,438.01
Chemical raw materials and chemical products manufacturing, rubber and plastic products industry	223,181	3.8781	10,356.64
Ferrous metal beneficiation, smelting and rolling processing industry	322,127	0.9469	7523.05
Paper and Paper Products Industry	172,710	13.7448	4142.72
Wood processing and wood, bamboo, rattan, palm, grass products industry	54,174	0.5471	3370.52
Electrical machinery and equipment manufacturing	42,763	0.2752	2960.14
Pharmaceutical manufacturing	33,062	0.6425	2684.75
Petroleum processing, coking and nuclear fuel processing industries	4735	0.0019	592.39
Culture and education, industrial art, sports, and entertainment products manufacturing, printing and recording media reproduction industry	10,467	0.5197	2159.11
Computer, communications and other electronic equipment manufacturing	6426	0.2875	1856.83
Special Equipment Manufacturing	4308	0.1362	1722.74
Metal products industry	9285	0.0295	1518.47

On the basis of the above data support, combined with the regional ecological development level, human development index, and other related measurement results, this study selected 15 candidate leading industries(P), namely: P1. breeding industry, P2. mineral resources development, P3. pharmaceutical manufacturing, P4. bamboo and timber planting and processing, P5. Tourism, P6. Chemical Industry, P7. Modern Trade and Commerce, P8. Financial Service Industry, P9. Paper Industry, P10. Digital Industry, P11. Modern Logistics Industry, P12. Electrical Machinery and Equipment Manufacturing, P13. Computer and other Electronic Equipment Manufacturing, P14. Cultural and Creative Industry, P15. Sustainable Energy Industry.

These 15 industries are chosen for the Huaihua City case study for sustainability because they were considered to be important for the city's economy and because they had the potential to be more sustainable. Agriculture, travel, manufacturing, IT, education, healthcare, building, transportation, energy, water, waste, and finance are all examples of these sectors.

Not every one of these sectors was a good fit for long-term viability. Agriculture and tourism, for example, have longstanding ties to environmental responsibility. Some sectors, including manufacturing and IT, may be more environmentally friendly with some adjustments to their operations. Several criteria were used to choose the sectors to examine in this case study. These included the sectors' relative significance to the city's economy, their potential to become more sustainable, and the quantity and quality of data available for analysis. Huaihua City's case study is an excellent tool for learning about sustainable development in China and the difficulties and possibilities it faces. The case study outlines many aspects crucial to attaining sustainable growth and offers insights into the sustainability-enhancing potential of various businesses.

#### Sustainable design-driven leading industry selection in Huaihua City

##### Sustainable design-driven leading industry selection mechanism

Rostow, Hirschman, and Shinohara standards are the most significant worldwide benchmarks for selecting top regional industries. China's regional economic leading industry selection process uses these three standards to construct three, four, five, six, and seven benchmarks and additional assessment criteria^42,43^. The "three benchmarks" and "five benchmarks" techniques of Zhou^44^ and Zhang^45^ are the most prominent. The "three benchmarks" method summarizes the selection criteria of leading industries based on their connotations and characteristics, while the "five benchmarks" method determines them based on their role, regional geographical information, and other characteristics. Income elasticity, productivity growth rate, industry correlation, coordination, technological upgrading, growth momentum, and competitiveness are benchmarks. This report selects and evaluates top industries using the "five benchmarks" technique. In addition, leading industries in ecologically sustainable development should be eco-friendly, technologically advanced, development-driven, environmentally adaptable, competitive, and risk-resistant^46–49^. After summarizing, this research presents sustainable design evaluation guidelines for selecting regional ecological leading sectors that combine ecological, cultural, and economic sustainability. These concepts should follow sustainable development principles of regional features, local citizens' acknowledgment, cultural integration, economic income growth, and local ecological industrial chains.

**Figure 4:**
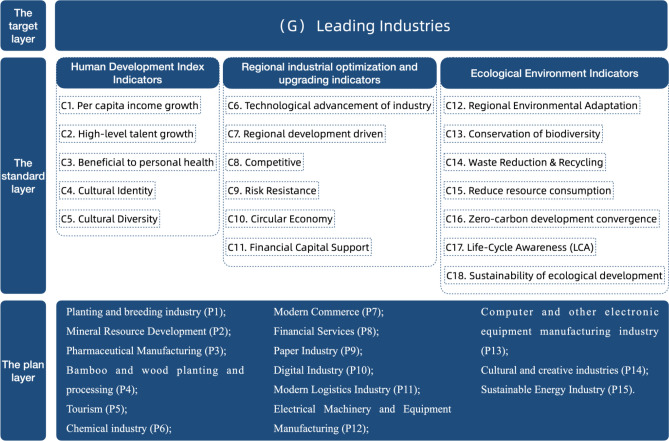
Model of dominant industry selection mechanism of regional eco-industry.

Hierarchical analysis was used to find regional eco-industry leaders (Fig. 4). The target layer (G) for the leading regional industry, the standard layer (C) for eco-industrial sustainable development, and the plan layer (P) for the candidate industry were the three layers of this mechanism. 18 qualitative indicators with three major directions of the human development index, regional industry optimization and upgrading index, and eco-industry index were created based on international and domestic research and sustainable industrial development factors. Each applicant industry has a guide layer (C) assessment index and score criteria from 1 to 9. Twelve relevant study professionals compared the tier criteria. MATLAB produced the judgement matrix and analyzed its greatest eigenvalues, eigenvectors, and consistency indices. After utilizing the consistency index to check that CR = 0.083464 was less than 0.1 and met the consistency criteria, the judgement matrix was assessed for reasonableness. Finally, regional ecological industry growth determines leading industry selection.

##### Leading industry selection in Huaihua City

After 12 experts gave their rankings of the 15 candidate leading industries according to the evaluation index system, the data were summarized, and the comprehensive evaluation values of the candidate industries were ranked (as shown in Table 8).

**Table 8:** Total utility value and ranking of candidate leading industries in Huaihua city.

Number	Industries	Total utility value
1	Planting and breeding industry (P1)	0.942233
2	Tourism (P5)	0.809222
3	Chemical industry (P6)	0.762619
4	Modern Commerce (P7)	0.742188
5	Pharmaceutical Manufacturing (P3)	0.722277
6	Mineral Resource Development (P2)	0.685421
7	Bamboo and wood planting and processing (P4)	0.652418
8	Modern Logistics Industry (P11)	0.602534
9	Computer and other electronic equipment manufacturing industry (P13)	0.592237
10	Digital Industry (P10)	0.587718
11	Sustainable Energy Industry (P15)	0.564379
12	Cultural and creative industries (P14)	0.519636
13	Paper Industry (P9)	0.423862
14	Electrical Machinery and Equipment Manufacturing (P12)	0.402350
15	Financial Services (P8)	0.379102

Huaihua's top five industries are P1. Modern ecological agriculture, P5. Intelligent cultural tourism, P6 chemical industry, P7 modern trade and commerce, and P3 pharmaceutical manufacture. These industries have geographical variances, complementarities, and synergies that follow the regional ecological industrial division of labor. They are supposed to generate synergistic regional economic growth and increase citizens' education, income, and happiness, increasing sustainable development. The chemical and mineral development industries, which make up a large part of the city's economy, are excluded from this paper due to their limited raw materials, ecological damage, and incompatibility with other leading industries in sustainable development.

Finally, using Huaihua's location, transportation, environment, cultural resources, and developed areas' industrial and economic activities, its key industries are: 1. modern ecological agriculture, relying on current agriculture conditions; 2. intelligent cultural tourism, relying on its rich cultural heritage; 3. pharmaceutical manufacturing, relying on natural ecological conditions; 4. digital economy, relying on modern trade and logistics; 5. electronic equipment manufacturing, relying on industrial transfer from Shenzhen and other developed regions. The five major sectors have regional variances, complementarities, and synergies that match the regional ecological industrial division of labor. By fostering synergistic economic growth and boosting citizens' education, income, and happiness, they can help maintain regional development.

#### Conceptualization of industrial synergy development mechanism based on leading industries with design intervention

**Figure 5:**
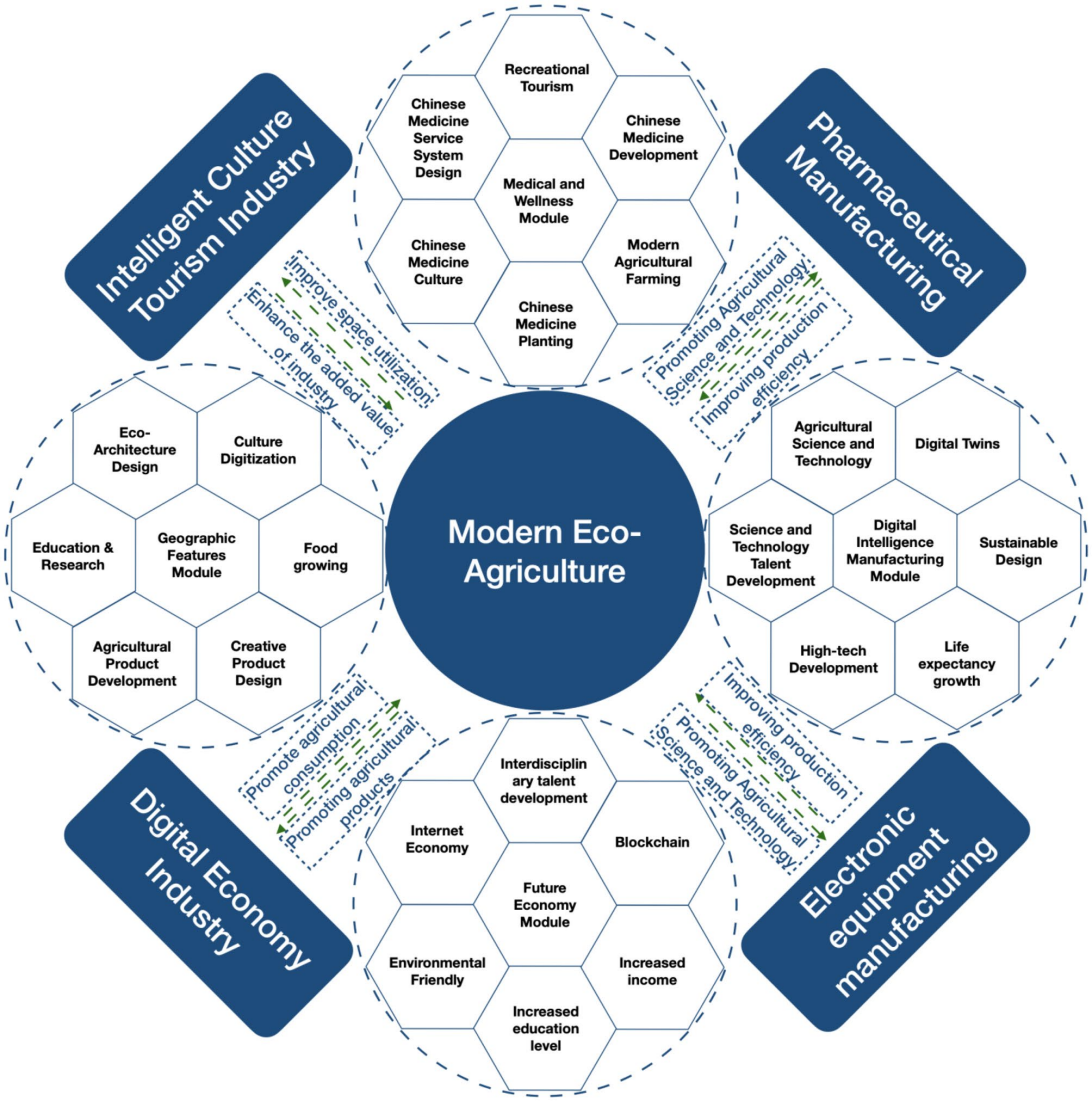
Sustainable development mechanism of synergistic symbiosis among leading industries with differentiation driven by design.

Figure 5 shows that the five top sectors have several synergies and complementarities from a sustainable design standpoint. Eco-smart agriculture may enhance regional spatial resource utilization, boost the value of modern agriculture, and reduce carbon emissions and ecological impact. Combining agriculture with ecological resources like "mountains, water, forests, fields, lakes, grass and sky" in the region will boost tertiary industries like education and research, recreation and leisure, and secondary industries like organic agriculture and food processing and manufacturing. The comprehensive promotion will boost the regional ecological agriculture industry and promote the new model of urban–rural integration under reverse urbanization in Huaihua City's modern trade and logistics industry. It develops agricultural science and technology, cultural creativity, and ecological architecture while strengthening the primary industry, generating strong links between leading industries and industrial sustainability^50,51^.

Although pharmaceutical, electronic product, and digital industries are relatively small, their development of high and new technology, senior talent training, regional cultural industry development, and ecological sustainability support the region's industrial upgrading. The aforementioned major industries may eliminate Huaihua's initial harsh industrial growth mode's environmental damage. Farming's environmental effect should still be considered. To reduce the ecological footprint of traditional fossil energy consumption in industrial development, improve regional spatial ecological welfare performance, and lay the industrial foundation for sustainable Huaihua City development, sustainable energy, and intelligent machinery and equipment should be actively developed and used.

Industry and practice affect the environment differently. Industrial environmental consequences include harmful effects of chemicals, heavy metals, and other contaminants that may damage water used by many businesses that is leading to air pollution, land pollution, resource depletion, and biodiversity loss. The case study selected prominent sectors based on their environmental implications. The case study excluded the most polluting sectors since they would need to adjust their practices to be sustainable. The case study highlights some sustainability issues and potential for these sectors. The case study shows how various sectors might become more sustainable and highlights key sustainability aspects.

- *The relevance of environmental impact assessment (EIA)*: EIAs identify and analyze a project or activity's possible environmental implications. EIA may assist in reducing the environmental consequences of projects and activities, making it a vital tool for sustainable development.
- *Government's involvement in sustainability*: Government can help promote sustainability. Governments may support sustainable enterprises and organizations via financial and technical aid and policy.

Huaihua can reconcile economic expansion with ecological protection by adopting many initiatives, including Encouraging sustainable industrial practices, Renewable energy, Protecting natural resources, and Public education. This case study represents these trade-offs. Agriculture and tourism are naturally sustainable. Manufacturing and IT can be more sustainable, but they need to evolve. Huaihua City's case study helps explain China's sustainable development difficulties and potential. The case study shows how various sectors might become more sustainable and highlights key sustainability aspects. Sustainable development requires stakeholder involvement. To create a community-supported sustainable development plan, all stakeholders must be consulted.

## Conclusions

Regional industry development and upgrading are unavoidable due to historical industrial evolution. Systematic design thinking will help select leading industries for sustainable regional industry transformation and build hierarchical analysis-based industrial clusters. This study chose Huaihua, a typical undeveloped city in the southwest of Hunan province, China. By examining Huaihua City's resources and industrial development, the sustainable development perspective targets the industrial transformation path and leading industries. At the same time, the leading industrial cluster configuration based on sustainable design effectively enhances regional industrial synergy and improves residents' living standards, which has important implications for southwest China's ecologically sustainable development.

This research examined design-driven techniques in Huaihua City, China. The findings may not apply to other Chinese or foreign cities directly. The case study only considers design's influence on sustainable development, not government policy or economic situations. The case study analyzed data from other organizations. The data may not have been acquired according to the case study's aims, which might skew the findings. Despite these limitations, this case study is useful for understanding China's sustainable development difficulties and prospects. The case study shows how design affects sustainable development and highlights key elements. Huaihua may have changed since the case study, thus the conclusions may not apply to other cities or countries. The case study solely addresses design's involvement in sustainable development which can be affected by government policies and economic circumstances.

## Data Availability

All data generated or analyzed during this study are included in this published article.
